# Glances at the Hospitals

**Published:** 1901-09-28

**Authors:** 


### GLANCES AT THE HOSPITALS.

### THE LEEDS GENERAL INFIRMARY.

The cover of this report tells us that the receipts, including legacies, amounted to £28,350; and the expenditure to £36,649, showing a deficiency of £8,299; but it ought to be stated that the ordinary expenditure amounted only to £27,108, which is, however, nearly £3,000 higher than it was during the previous year. No hospital should, or can, go on indefinitely spending a larger sum than it receives, and if the board of management of the Leeds Infirmary cannot increase their income it is incumbent on them to reduce their expenditure. The medical, surgical, and operation tables leave nothing to be desired. The numbers afflicted by any diseases can be easily followed to their ending in recovery, relief or death. For instance, we notice that there were seven cases of diphtheria, of whom five recovered and two died. There were 36 cases of strangulated hernia, and of these 26 recovered after operation and 10 died. The operation for the radical cure of hernia was performed 98 times and every case was successful. Anæsthetics were used 3,898 times. Ether was the agent in 1,505 cases, chloroform in 428, A.C.E. mixture in 299, C.E. mixture in 18, and nitrous oxide in 34. The rest were one or more of the above agents followed by a second or third. The total capacity of the infirmary is 436 beds, and the semi-convalescent hospital contains 46 beds. The total cost up-to-date has been £227,439. During the year 6,071 new in-patients were admitted; and 31,961 new out-patients were treated. Mr. Benson Jowitt resigned the office of treasurer to the infirmary, and it was decided to mark his services by having his portrait painted and placed in the board room, and to establish a pension fund for nurses to be known as "The Jowitt Pension Fund."

### SHEFFIELD HOSPITALS FOR INFECTIOUS DISEASES.

At the end of the year there remained 232 cases in these hospitals, and 1,475 were admitted during the year. Of these 777 were scarlet fever, 108 were enteric, 571 were diphtheria, and 19 were described as "other cases." Scarlet fever shows a mortality of 3·2 per cent. The most frequent complication was otitis media, from which 89 suffered; secondary adenitis affected 69 cases, and albuminuria 49. Altogether 352 out of a total of 796 had complications, so that the mortality must be looked on as a low one. The pressure on the wards for enteric fever was so great that only 21 per cent. of the cases reported could be admitted. This percentage represented 108 cases, and with those remaining from the previous year made a total of 128 under treatment—all these being urgent cases. The mortality was 11·8 per cent. As complications the most frequent were pneumonia, venous thrombosis, intestinal hæmorrhage and nephritis, these being found respectively in 5, 4, 4 and 3 instances. Of the 108 admissions, 80 were between the ages of five and 30, and there were only 10 cases over 40 years of age. The highest death-rate was between the ages of 10 and 15. As already stated the diphtheria cases numbered 571. Of this number 160 were under five years of age, and 38 died, showing a mortality of 23·7 per cent. Between five and 10 years of age there were 199 cases, and 35 died, showing a mortality of 17·6 per cent. Above the age of 10 the mortality steadily diminished. Of 45 cases between 15 and 20 there was no death; of 76 over 20 there were two deaths. Taking all ages the mortality was 13·7 per cent. Albuminuria occurred in 41·1 per cent. of the patients, and paralysis in 12·9 per cent. The experience obtained by these 571 cases of diphtheria illustrates in the most forcible manner the immense importance of early treatment, for of 124 cases admitted during the first three days of the attack there was only one death. Among the "other cases" we notice pneumonia, with which disease seven cases were admitted; tonsillitis appeared in three and ulcerative colitis in one. Dr. Hardy's report is a model of what such documents should be; and the accompanying tables are drawn with much clearness. Considerable additions are being built to these hospitals.

### THE GUEST HOSPITAL, DUDLEY.

The committee regret to report a further falling off in the annual subscriptions, and they attribute it to the fact that there are now two hospitals in the districts, and that many of their old subscribers have died. The committee further say, and we desire to give their words publicity, that "it might reasonably be expected that in a great commercial centre the private subscriptions would be much more satisfactory. An earnest appeal is made to all who are in a position to exercise a charitable spirit." It is a pleasing feature that the workmen's subscriptions amounted to £767; but nevertheless that sum is lower by several pounds than it was the previous year. The Sunday collections improved by nearly £70. Still there was an adverse balance of £79 on the year's working, which added to that of the previous year makes a total deficit of £246. The average cost per bed occupied was £53, and the average stay of patients in the wards was 4½ weeks. The total number of patients was 784. Of these 620 were discharged cured or relieved, 28 were incurable, 15 were discharged at own request, and 64 died; but of the last number 23 were moribund on admission. The committee may look on the matter in another light, but we think it a hopeful sign for the medical profession that the applications for the posts of resident medical officers are not so numerous as they were a few years ago, neither at the Guest Hospital nor elsewhere, and at this hospital they intend throwing open the junior appointments to ladies. The operation table is a long one and it is carefully and properly compiled. Everything necessary connected with the operation is stated: the nature of it, the sex and age of patient, the date of operation, the result, and "remarks" are given in admirable order. The same may be said of the post-mortem table. And these facts make it all the more wonderful that the ordinary medical and surgical tables merely name diseases and give numbers of patients. We should also like to see an anæsthetic table in the next report.

### THE MANCHESTER SOUTHERN HOSPITAL FOR WOMEN AND CHILDREN.

Here the total income was £2,419, and the total sum expended was £3,374. The latter included repairs and renewals; but, however explained or qualified, there was a deficit of £955 on the year's working. The patients contributed £249 towards the income. The medical report occupies barely four pages. We learn that there were 1,360 out-patients, and 137 in-patients. The operations numbered 137, and there were 7 deaths; but which operations were followed by these deaths we do not learn. In the maternity department 183 patients were treated, and 60 of these were abnormal cases, there being 2 deaths—1 from eclampsia and 1 from accidental hæmorrhage. In the children's department there were 2,699 out-patients, and 213 in-patients. Of the latter 53 were medical, and 159 surgical. The operations in this department numbered 208, the most of them being minor. Altogether there were 8 deaths among the children, 6 being caused by pneumonia, 1 by malnutrition, and 1 by peritonitis.

### THE CROYDON GENERAL HOSPITAL.

The income for the past year was £4,482, and the expenditure was £4,427, showing a balance to the good of £55; but the committee truly enough say that with the opening of the Jubilee wing the number of patients will increase and more money will be required to carry on the useful work of the hospital. The new wing with its furnishing and offices cost £11,258, and it was formally opened by the Princess Christian. The year has been a memorable one, and it is not likely that the people of Croydon will fail to respond to the wishes of the Board of Management for an increase in the annual subscriptions. Indeed, we see that Mr. Edward A. Ray has presented £1,000 to the reserve fund; Hospital Sunday produced £703; and Hospital Saturday £640. At the beginning of the year there were 41 patients in the hospital, and 871 were admitted during the year, making a total of 912. Of those admitted 457 were men and 414 women. The medical and surgical report is very meagre and is useless for purposes of comparison and almost useless for obtaining any information from it, as merely the numbers suffering from the diseases are given. For instance, we notice that 27 cases of gastric ulcer were treated during the year, but we hear nothing of the results. On turning to the table showing the causes of death we find that gastric ulcer appears twice, so that we may assume that 25 cases recovered. Without reckoning dental cases there were 6,619 individuals treated in the out-patients' department.

